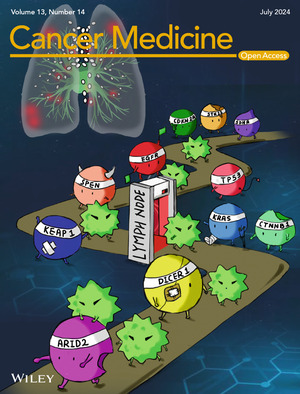# Cover Image

**DOI:** 10.1002/cam4.70116

**Published:** 2024-08-29

**Authors:** Wei Guo, Tong Lu, Yang Song, Anqi Li, Xijia Feng, Dingpei Han, Yuqin Cao, Debin Sun, Xiaoli Gong, Chengqiang Li, Runsen Jin, Hailei Du, Kai Chen, Jie Xiang, Junbiao Hang, Gang Chen, Hecheng Li

## Abstract

The cover image is based on the article *Lymph node metastasis in early invasive lung adenocarcinoma: Prediction model establishment and validation based on genomic profiling and clinicopathologic characteristics* by Wei Guo et al., https://doi.org/10.1002/cam4.70039.